# Effectiveness of a Tiered Referral System and Early Nutritional Intervention to Prevent and Recover Stunting in Under‐Five Indonesian Children

**DOI:** 10.1002/fsn3.70945

**Published:** 2025-10-23

**Authors:** Damayanti Rusli Sjarif, Klara Yuliarti, Lanny Christine Gultom, Cut Nurul Hafifah, I. Gusti Lanang Sidiartha, Meta Herdiana Hanindita, Neti Nurani, Aidah Juliaty, Ali Alhadar, Anik Puryatni, Arief Budiarto, Endy Paryanto Prawirohartono, Irma Sri Hidayati, Julius Anzar, Moretta Damayanti, Nur Aisiyah Widjaja, Nice Rachmawati Masnadi, Rina Pratiwi, Rini Andriani, Ronald Rompies, Novitria Dwinanda, Winra Pratita, William Jayadi Iskandar, Jessy Hardjo

**Affiliations:** ^1^ Department of Child Health, Faculty of Medicine Universitas Indonesia/Dr Cipto Mangunkusumo Hospital Jakarta Indonesia; ^2^ Department of Child Health Fatmawati General Hospital Jakarta Indonesia; ^3^ Department of Child Health, Faculty of Medicine Universitas Udayana/Sanglah Hospital Denpasar Indonesia; ^4^ Department of Child Health, Faculty of Medicine Universitas Airlangga/Dr Soetomo Hospital Surabaya Indonesia; ^5^ Department of Child Health, Faculty of Medicine, Public Health and Nursing Universitas Gadjah Mada/Dr Sardjito Hospital Yogyakarta Indonesia; ^6^ Department of Pediatrics, Faculty of Medicine Hasanuddin University/Dr Wahidin Sudirohusodo Hospital Makassar Indonesia; ^7^ Department of Child Health Hermina Jatinegara Hospital Jakarta Indonesia; ^8^ Department of Pediatrics, Faculty of Medicine Universitas Brawijaya/Saiful Anwar Hospital Malang Indonesia; ^9^ Department of Pediatrics, Faculty of Medicine Lambung Mangkurat University/Ulin Banjarmasin Hospital Banjarmasin Indonesia; ^10^ Department of Pediatrics, Faculty of Medicine Universitas Sriwijaya/Dr Mohammad Hoesin General Hospital Palembang Indonesia; ^11^ Department of Pediatrics, Faculty of Medicine Universitas Andalas Padang Indonesia; ^12^ Department of Pediatrics, Faculty of Medicine Universitas Diponegoro/Dr Kariadi Hospital Semarang Indonesia; ^13^ Department of Pediatrics Medical Faculty of Tanjungpura University/Kharitas Bhakti Hospital Pontianak West Kalimantan Indonesia; ^14^ Department of Pediatrics National Women and Children Health Center RSAB Harapan Kita Jakarta Indonesia; ^15^ Department of Pediatrics, Faculty of Medicine Universitas Sumatera Utara Medan Indonesia

**Keywords:** catch‐up growth intervention, dietary animal proteins, FSMP, patient education, stunting

## Abstract

Stunting remains a major global malnutrition problem in children. A protocol for stunting prevention and management in Indonesia is needed to achieve the WHO's stunting reduction target by 40% in 2025. This study aimed to evaluate the effectiveness of a tiered referral system across different healthcare levels and the importance of animal proteins and food for special medical purposes (FSMP) in preventing and managing stunting in Indonesia. This one‐group pre–post experimental study involved children under 5 years old from 14 regencies in Indonesia. All subjects initially received education about breastfeeding and animal proteins. Eligible subjects were then given daily animal protein (milk and/or egg) supplementation. Subjects with weight faltering, underweight, or wasting were referred and given nutritional intervention for 2 weeks. If treatments failed to normalize undernutrition, subjects were referred to regional hospitals. Those confirmed as stunted or with low birth weight were directly referred for treatment and FSMP as indicated. A total of 1841 subjects received egg and/or milk supplementation. In 6 months, animal protein supplementation prevented weight faltering in 1320/1841 subjects (71.7%; 95% CI 69.6%–73.8%). Red flags management successfully prevented stunting in 536/709 subjects (75.6%; 95% CI 72.4%–78.8%) in 2 weeks. FSMP prescription for catch‐up growth in regional hospitals led to stunting recovery in 166/381 subjects (43.6%; 95% CI 38.6%–48.5%) in ±14 weeks. A tiered referral system with well‐defined treatment guidelines is crucial to address stunting in Indonesia. Timely referral and FSMP prescription for catch‐up growth can effectively treat stunting. Animal proteins and education are keys to preventing stunting in children.

## Graphical abstract

Protein‐based supplementation at the community level (e.g., milk and/or egg) prevented weight faltering in 71.7% of children. Early diagnosis and timely treatment in primary healthcare services prevented stunting in 75.6% of cases. Among stunted children referred to regional hospitals, 43.6% recovered within 6 months with FSMP for catch‐up growth. This comprehensive strategy, along with proper education at all levels of care, offers an effective protocol for stunting prevention and management.

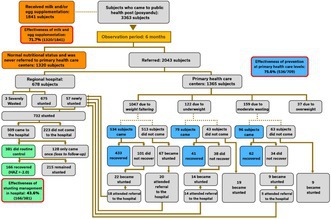

## 1 Introduction

Stunting remains a major global health problem, especially in developing countries. Stunting is defined as children with a height/length‐for‐age *z*‐score less than −2 standard deviations (SD) for their respective age and sex, according to the World Health Organization (WHO) 2006 standard growth chart (World Health Organization 2014a). Stunting results from chronic or recurrent undernutrition and is commonly associated with poor socioeconomic conditions, inadequate maternal health and nutrition, frequent illnesses, and/or inappropriate infant and young child feeding practices during a child's early years (Fatima et al. 2020; Yani et al. 2023). The consequences of stunting extend beyond mere physical growth retardation. It impairs cognition, memory, and attention, leading to poor academic achievement and reduced future potential (De Sanctis et al. 2021). Children with stunting are also more susceptible to infections and chronic diseases, poorer school performance, and lower economic productivity in adulthood (de Onis and Branca 2016). The global prevalence of stunting in 2022 was 22.3% (UNICEF 2023). In Indonesia, the prevalence has declined from 37.2% in 2013 to 21.6% in 2022 (Ministry of Health Republic of Indonesia 2022a). However, 18 of 34 provinces had stunting prevalence exceeding the national average (Ministry of Health Republic Indonesia 2022a). Despite the improvement, prompt actions are needed to meet the stunting prevalence target of 14% for 2024 set by the Indonesian government (Ministry of Health Republic Indonesia 2020), which aligns with the WHO's global goal of a 40% reduction in stunting incidence by 2025, equivalent to a 3.9% annual reduction (World Health Organization 2014b).

Numerous global studies have established a link between children's linear growth and proteins from animal sources, such as milk and eggs (Inzaghi et al. 2022; Uauy 2013; Xiong et al. 2023). Animal proteins have a more significant impact on linear growth compared to plant‐based proteins because they contain more complete essential amino acids and sulfur‐ring amino acids (Wu 2010). However, effective stunting reduction requires not only prevention of new stunting cases but also treatment of existing ones. The treatment for stunted children in Indonesia falls within a pediatrician's competence, regulated in the national stunting management guidelines (Ministry of Health Republic Indonesia 2022b).

Healthcare services in Indonesia are classified into three levels: primary, secondary, and tertiary (Werdhani 2019). This study aimed to evaluate the implementation of a well‐defined, tiered referral system involving the three healthcare levels to prevent and manage stunting problems in Indonesia. To the best of our knowledge, no studies in Indonesia have investigated the role of a comprehensive referral system as part of stunting management strategies. In addition, the effectiveness of animal proteins for stunting prevention and FSMP for catch‐up growth as part of stunting treatment was also assessed in this study.

## 2 Methods

### 2.1 Settings

**FIGURE 1:**
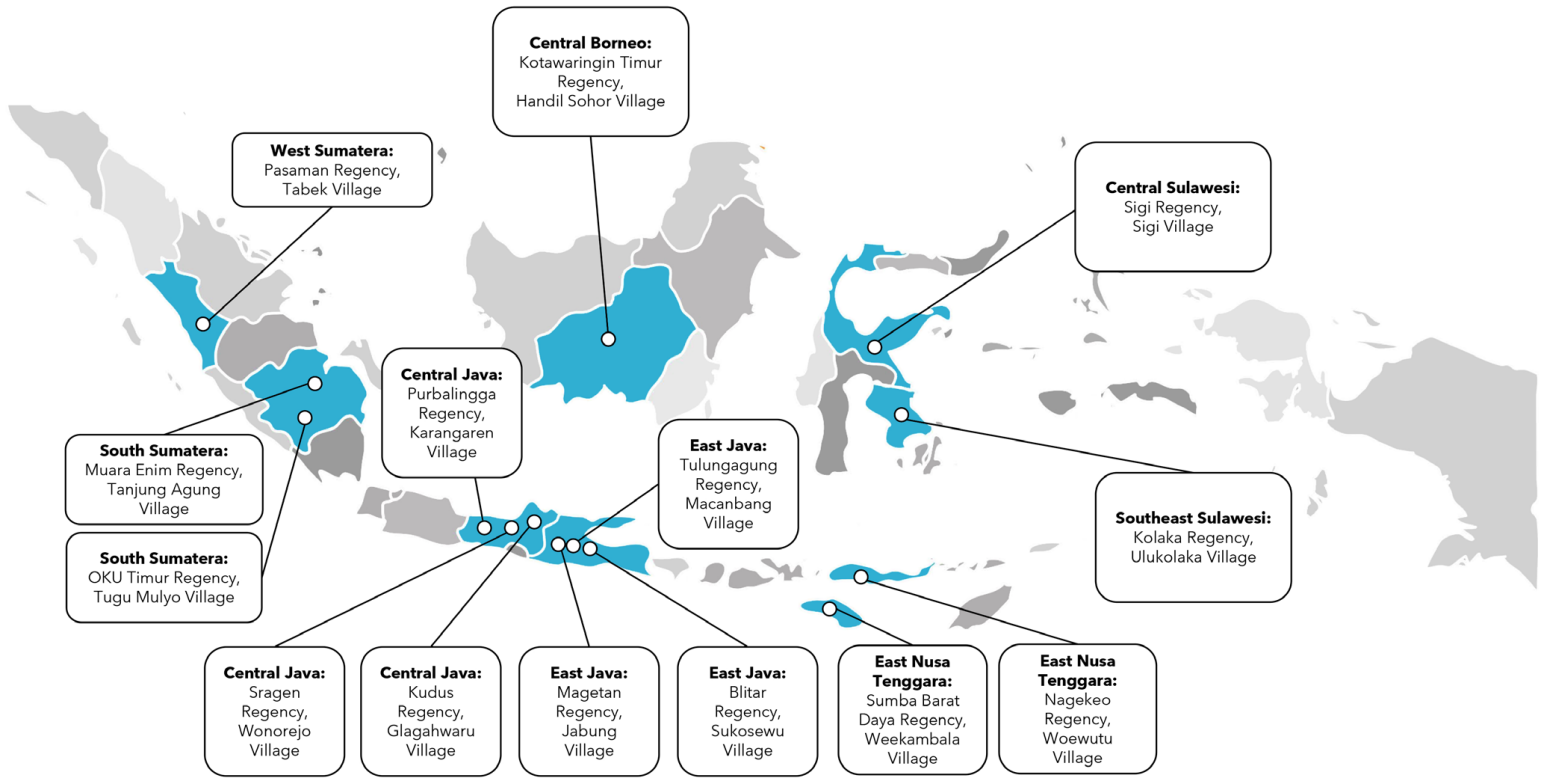
Study areas all over Indonesia, involving 14 villages across 14 regencies. West Sumatera: Pasaman Regency; South Sumatera: Muara Enim Regency and Ogan Komering Ulu (OKU) Timur Regency; Central Java: Kudus Regency, Purbalingga Regency, and Sragen Regency; East Java: Magetan Regency, Tulungagung Regency, and Blitar Regency; East Nusa Tenggara: Nagekeo Regency and Sumba Barat Daya Regency; Central Borneo: Kotawaringin Timur Regency; Central Sulawesi: Sigi Regency; Southeast Sulawesi: Kolaka Regency.

The study was conducted from November 2020 to September 2022 across 14 regencies in Indonesia (Figure 1, details in Table S1). These regencies were selected from 43 regencies that applied, based on two criteria: (1) having a local team comprised of a pediatrician, general practitioner, midwife, nutritionist, cadres, village head, and Family Welfare Empowerment Program (*Pemberdayaan Kesejahteraan Keluarga*); and (2) willingness to create a report and join discussions every month.

**FIGURE 2:**
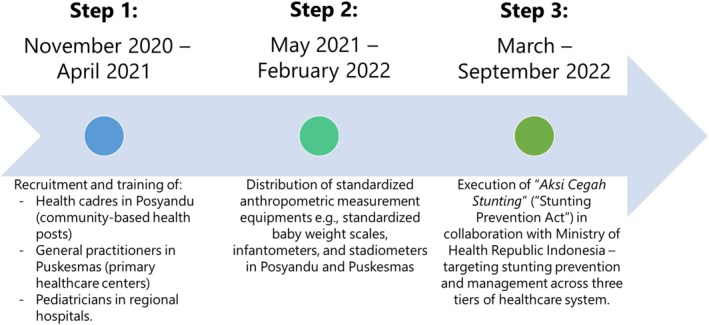
Study steps, involving recruitment and training of health cadres and other healthcare professionals, distribution of supporting tools, and execution of targeted stunting management. During step 1, recruitment and training of health cadres, general practitioners, and pediatricians were performed in each regency and villages. During step 2, provision of standardized anthropometric measurements by Ministry of Health Republic Indonesia included standardized baby weight scales (LAICA Baby Scale BF2051, precision of 10 g), infantometers (SECA Model 417, precision to 0.1 cm), and stadiometers (Portable Stadiometer METRISIS in Posyandu and SECA Stadiometer Model 213 in Puskesmas, both with precision to 0.1 cm). During step 3, all research activities were monitored and supervised via online meeting throughout the study. The “Aksi Cegah Stunting” facilitators were pediatric nutrition and metabolic disease consultants in each district, supervised by consultants from Jakarta and local district.

The study was divided into three steps: resource training, equipment preparation, and the main intervention (Figure 2). The main intervention program, named “Stunting Prevention Act” or “Aksi Cegah Stunting (ACS)” in Indonesian language, involved three tiers of healthcare centers in Indonesia, which are Posyandu, Puskesmas, and regional hospitals. Posyandu are community‐based centers that are run by trained health cadres to provide maternal and child health services (Leimena 1989). Meanwhile, Puskesmas refer to primary healthcare centers in Indonesia, which are served by general practitioners, dietitians, and nurses/midwives. A consistent referral pathway of Posyandu–Puskesmas–regional hospitals was implemented based on the Regulation of the Minister of Health, Republic of Indonesia No. HK.01.07/MENKES/1928/2022 on National Guidelines for Medical Services in Stunting Management (Ministry of Health Republic Indonesia 2022b).

All children visiting Posyandu during the study period were included in this study, except children aged > 60 months during the program, refused to participate, or with syndromic/neurologic disorders such as cerebral palsy. The characteristics of the study subjects are shown in Table 1. All regencies utilized standardized anthropometric measurements, in accordance with the criteria set forth by the WHO and Regulation of the Minister of Health, Republic of Indonesia (*Keputusan Menteri Kesehatan Republik Indonesia No*. HK.01.07/MENKES/1919/2022) (Ministry of Health Republic Indonesia 2022c). Standardized baby weight scales (LAICA Baby Scale BF2051, precision of 10 g), infantometers for subjects ≤ 2 years old (SECA Model 417, precision to 0.1 cm), and stadiometers for subjects aged 2–5 years old (Portable Stadiometer METRISIS in Posyandu and SECA Stadiometer Model 213 in Puskesmas, both with precision to 0.1 cm) were all supplied by the Indonesian Ministry of Health.

**TABLE 1:** Characteristics of the study subjects at baseline.

Characteristics	Subjects, *N* (%)
Age	
< 6 months	246 (7.3)
6–23 months	1085 (32.3)
24–60 months	2032 (60.4)
Sex	
Male	1748 (51.9)
Female	1615 (48.1)
Nutritional status at start	
Normal	1841 (54.7)
Underweight	807 (24.0)
Severe wasting	3 (0.1)
Stunting	675 (20.1)
Overweight	37 (1.1)
Subject distribution	
Pasaman	118 (3.5)
Blitar	837 (24.9)
Tulungagung	138 (4.1)
Magetan	219 (6.5)
Kolaka	266 (7.9)
Muara Enim	344 (10.2)
Ogan Komering Ilir Timur	259 (7.7)
Purbalingga	119 (3.5)
Sragen	223 (6.6)
Nagekeo	56 (1.7)
Sumba Barat Daya	145 (4.3)
Kudus	247 (7.3)
Sigi	247 (7.3)
Kotawaringin	145 (4.3)

### 2.2 Education and Daily Milk and Egg Supplementation

In the beginning of the study and during every Posyandu visit, parents or caregivers were educated about infant and young child feeding with special emphasis on providing optimal breastfeeding and adequate local animal source protein in daily complementary feeding or family food. Details of recommended daily protein intake and food types were described in Table 2 (Ministry of Health Republic Indonesia 2019; World Health Organization 2000). To help fulfill the protein RDA, this study supplemented children with local animal‐source foods that could provide almost 50% of the RDA. A box of 110 to 125 mL plain UHT milk contains approximately 4 g of protein, and one chicken egg contains around 6 g of protein. Therefore, children aged 6–11 months old were given one chicken egg (±6 g of proteins per day), children 12–23 months old were given one egg and one milk (a total of ±10 g of proteins per day), and children ≥ 24 months were given three boxes of milk (±12 g of proteins per day), as shown in Table 3.

**TABLE 2:** The recommended daily protein intake and types of food for each age group.

Age	Daily protein intake (g)^a^	Energy needed from complementary/family food (kcal)^b^	Portion of animal protein source of total calories (%)^c^	Protein‐energy ratio (%)	Example of food^d^	Types of supplementation given in this study
6–8 months (breastfed)	15	200	30	9	1 chicken egg (protein 6 g)^b^	1 chicken egg
9–11 months (breastfed)	15	300	50	10	1 chicken egg + ½ chicken liver (protein 3 g)	1 chicken egg
12–23 months (breastfed)	20	550	70	10	1 chicken egg + 30 g mackerel (protein 6 g) + 1 box of 125 mL plain UHT milk (protein 4 g)	1 chicken egg and 1 box of plain UHT milk (110–125 mL)
24–60 months	25	1400	100	7.1	2 chicken eggs + 1 chicken liver OR 30 g red meat (protein 6 g) + 2 boxes of UHT 125 mL OR 30 g anchovy + 2 chicken eggs + 3 boxes of UHT 125 mL	3 boxes of plain UHT milk (110–125 mL)

^a^ Based on the Indonesian recommended daily allowance (Ministry of Health Republic Indonesia 2019).

^b^ Based on the WHO guideline for infant feeding practices (World Health Organization 2009).

^c^ Based on protein deficit during breastmilk consumption (USDA Food Data Central n.d.).

^d^ Depends on the local availability of animal protein sources.

**TABLE 3:** Animal protein interventions given according to the children's age.

Age (months)	Protein intervention (per day)	Education given to parents about main protein sources for daily complementary feeding
6**–**11	1 chicken egg	Incorporate locally available animal proteins in daily complementary foods, using varieties such as: 1 tablespoon of minced beef, fish, or chicken ½ to 1 tablespoon of chicken liver 1 egg
12**–**23	1 chicken egg and 1 box (110–125 mL) of plain, no added sugar ultra‐high‐temperature (UHT) milk	Incorporate locally available animal proteins in daily complementary foods, using varieties such as: 1 chicken liver 1 egg and 1 tablespoon of minced beef, fish, or chicken 1 egg and 1 box of UHT milk
24**–**59	3 boxes (each 110–125 mL) of plain, no added sugar UHT milk	Incorporate locally available animal proteins in daily complementary foods, using varieties such as: 1 egg and 1 chicken liver 1 egg and 3 tablespoons of minced beef, fish, or chicken 2 eggs

Animal protein supplementation (milk and/or eggs) was exclusively given to subjects who were born at term, had normal birth weight, appeared clinically healthy, and had normal anthropometric measurements in WHO growth charts. Subjects who did not fulfill these criteria were referred to Puskesmas. The milk and/or eggs were distributed weekly in conjunction with routine weight measurements at Posyandu. Supplementation was continued for another week if subjects gained adequate weight (> 100 g/week for 6‐ to 11‐month‐old infants, or > 50 g/week for 12‐ to 59‐month‐old children) and stopped if subjects did not, and then referred to Puskesmas.

### 2.3 Healthcare Referral System for Malnutrition

Upon referral to Puskesmas, subjects were evaluated by a trained general practitioner (GP), and anthropometric measurements were re‐measured. Subjects with weight faltering, underweight, and wasting were treated in Puskesmas. The GP performed assessments for red flags, such as the tuberculin test to screen for tuberculosis and urinalysis to screen for urinary tract infection, and subjects were treated accordingly as indicated. For catch‐up growth, subjects were given special dietary intervention at 30% of their total calorie requirement for 2 weeks, in addition to their normal diet consisting of animal proteins. The special dietary formula was formulated based on the Community Management of Acute Malnutrition (CMAM) recommendation that fits the Codex Alimentarius composition for infant formula and growing‐up formula. Registered and approved by the National Agency of Drug and Food Control in Indonesia, the special dietary formula was composed of animal‐source protein with a protein energy ratio (PER) > 10%, no added or added sugar < 10%, and was prescribed and supervised by the GP. Subjects in Puskesmas also received education from the GP about feeding rules and the importance of animal protein sources in each meal, along with the special dietary intervention.

Subjects were sent back to Posyandu if they achieved adequate weight gain, were of normal weight, and had normal nutritional status. Those with inadequate response after 2 weeks or had unimproved red flag conditions were referred to regional hospitals. Subjects with severe acute malnutrition were given F100 formula and mineral mix following the WHO guidelines. If F100 was unavailable, subjects were referred to the regional hospital. To conclude, subjects were referred to the regional hospital if: (1) absence of improvement in nutritional status or presence of an underlying disease after 2 weeks of treatment, (2) subjects had diseases beyond the competence of a general practitioner, and (3) confirmed to be stunted or had low birth weight.

At regional hospitals, trained pediatricians assessed subjects with short stature to determine if the cause was stunting or other factors. Evaluations included dietary history, weight and length measurements, physical examination, and red flag assessments. Stunting was diagnosed if weight and length increments were below normal or if weight age was lower than height age. For children ≥ 2 years old, bone age assessment helped differentiate familial short stature from constitutional growth delay. Mid‐parental height was also calculated to assess genetic influences. Laboratory examinations to evaluate red flags were conducted as needed, such as the tuberculin test for tuberculosis screening. All stunted children received Food for Special Medical Purposes (FSMP) in the form of oral nutritional solutions (ONS) in accordance with the prescription guidelines presented in Table 4.

**TABLE 4:** Food for special medical purpose (FSMP) prescription for stunting.

No	Age group (months)	Category	Oral nutrition solution (ONS)	Requirement (mL/day)	Duration of prescription per visit^a^(day)
1	0–12	Stunting + severe wasting (WHZ < −3)	ONS 1 kcal/mL	800	14
2	0–12	Stunting + moderate wasting (−3 < WHZ < −2)	ONS 1 kcal/mL	500	14
3	0–12	Stunting + normal WHZ + underweight (WAZ < −2)	ONS 1 kcal/mL & PER ≥ 10%	500 (4 bottles)	14
4	6–12	Stunting + normal WHZ + normal body weight (WAZ ≥ −2)	ONS 1 kcal/mL & PER ≥ 10%	500 (4 bottles)	30
5	> 12	Stunting + severe wasting (WHZ < −3)	ONS 1.5 kcal/mL	800 (4 bottles)	14
6	> 12	Stunting + moderate wasting (−3 < WHZ < −2)	ONS 1.5 kcal/mL	600 (3 bottles)	14
7	> 12	Stunting + normal WHZ + underweight (WAZ < −2)	ONS 1 kcal/mL & PER ≥ 9%–10%	500	14
8	> 12	Stunting + normal WHZ + normal body weight (WAZ ≥ −2)	ONS 1 kcal/mL & PER ≥ 10%	750	30

Abbreviations: PER, protein energy ratio; WAZ, weight‐for‐age z‐score; WHZ, weight‐for‐length/height z‐score.

^a^ Maximum duration of treatment is 6 months in this study.

Subjects with food allergies, preterm births, or inborn errors of metabolism received FSMP according to their underlying disease, including human milk fortifier, premature formula, amino‐acid‐based formula, or formula for inborn errors of metabolism. All subjects received outpatient treatment at respective regional hospitals until they recovered from stunting and attained normal anthropometric measurements (HAZ ≥ –2, WHZ ≥ –2, and WAZ ≥–2). Subjects who reached normal nutritional status were referred back to Puskesmas. (Ministry of Health Republic Indonesia 2022b).

### 2.4 Monitoring

Facilitators, consisting of pediatric nutrition and metabolic disease consultants, monitored patient progress, ensured measurement accuracy, oversaw referrals, and collaborated with the local ACS team to address challenges. The local ACS team included local government authorities, provincial health offices, village midwives, general practitioners, pediatricians, cadres, and field nutritionists. A monthly online meeting was held with consultant pediatricians, local pediatricians, general practitioners, dietitians, midwives, and cadres. All distribution of anthropometric measurement tools, eggs and milk boxes, formula milk in Puskesmas, and FSMP in regional hospitals was provided by the Indonesian Ministry of Health and distributed through the provincial health offices.

### 2.5 Data Analysis

The WHO Anthro software was used to calculate z‐scores. Children were categorized as stunted if their HAZ score was below −2 SD, underweight if their WAZ was below −2 SD, and wasted if their WHZ was below −2 SD. Severe cases were defined as z‐scores below −3 SD. Descriptive analysis was conducted for HAZ, WAZ, and WHZ by comparing the mean values at baseline and after the intervention to observe trends in nutritional status. The effectiveness of milk and egg supplementation was measured by comparing the number of healthy subjects after 6 months to the number of all included subjects at the beginning. The effectiveness of primary health care services was assessed by the number of recovered subjects in Puskesmas divided by the total number of subjects who responded to the referral and came to Puskesmas. The effectiveness of stunting management was calculated by the number of recovered subjects at regional hospitals divided by the total number of subjects who complied with the referral and came to the regional hospitals. Baseline stunting prevalence in the 14 regencies was compared to the endline prevalence, and the significance of stunting decline was analyzed using the Chi‐square test. The correlation between Posyandu visit frequency and stunting reduction was analyzed using the Chi‐square test at a 95% confidence interval. Visit frequency was grouped into 2–3 times, 4–5 times, and 6–7 times. Stunting reduction was defined as a decrease in prevalence from baseline to endline. Odds ratios (ORs) were calculated as crude ORs using 2 × 2 contingency tables to estimate the likelihood of stunting reduction (OR a–b) and new stunting cases (OR a–c) across Posyandu visit frequency categories. No multivariate adjustments were made. The dependent variable was binary, representing either the presence or absence of stunting at endline, and the reference category was the group with 2–3 visits. All analyses were performed using IBM SPSS Statistics (version 26) with statistical significance set at *p* < 0.05.

### 2.6 Ethics Statement

This study protocol was reviewed and approved by the Ethical Committee of Health Research, Faculty of Medicine Universitas Indonesia, approval number KET‐388/UN2.F1/ETIK/PPM.00.02/2022. Written informed consent was obtained from parents or legal guardians at the beginning of the study. All data were managed in accordance with human subject research and data confidentiality.

## 3 Results

**FIGURE 3:**
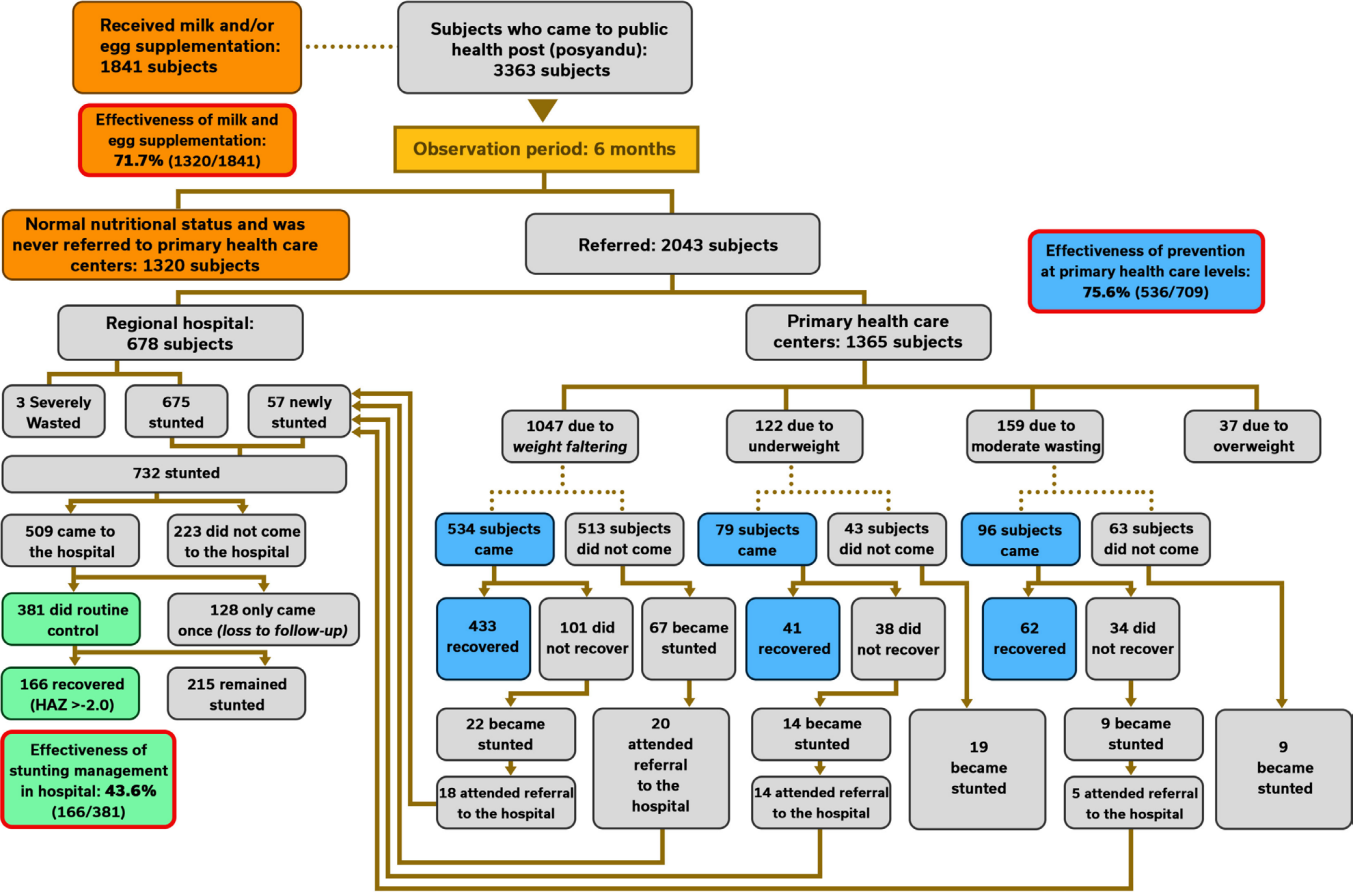
Flowchart depicting the tiered referral system implemented in the study. Initial observation period was 6 months. Routine visit to Puskesmas/regional hospital was scheduled at least twice every 1 or 2 weeks. In regional hospitals, full stunting recovery was observed in an average of 3.4 months. HAZ, height‐for‐age *z*‐score.

A total of 3363 subjects from 14 regencies were included in this study. The characteristics of study subjects are portrayed in Table 1, and the overall flowchart of the study referral protocol is depicted in Figure 3. Among these, 1841 subjects had normal anthropometric measurements at Posyandu and received milk and egg protein supplementation in March 2022, as described in Table 3. From this cohort, 1320 subjects had normal nutritional status and continued to receive the supplementation for 6 months. The remaining subjects were referred to Puskesmas at some point during the study (Figure 3). The intervention demonstrated that the effectiveness of milk and egg as a primary prevention of stunting was 71.7% during a 6‐month period of observation.

A total of 2043 subjects were referred to Puskesmas, with 1365 subjects referred for conditions such as weight faltering, underweight, moderate wasting, or overweight. The remaining 678 subjects were directly referred to regional hospitals, 675 due to stunting and three due to severe wasting. Among the 1365 referred subjects, 709 (51.9%) came and sought treatment at Puskesmas, and 536 subjects successfully recovered and were sent back to Posyandu. The effectiveness of stunting prevention through early detection and management of red flags at the primary healthcare level was 75.6%. However, 170 subjects failed to recover, and 44 of 706 subjects (6.2%) became stunted. These newly stunted subjects were referred to the regional hospital. Meanwhile, there were 619 of 1365 subjects (45.4%) who were referred but refused to come to Puskesmas. From these 619 subjects who did not seek treatment, 95 (15.3%) developed stunting.

Among 732 subjects with stunting who were referred to regional hospitals, 509 subjects (69.5%) came to the hospital. A total of 381 of 509 subjects (74.9%) routinely visited the hospital at least twice every 1 or 2 weeks (according to the protocol depicted in Figure 3), whereas the remaining 128 subjects (25.1%) only visited the hospital once and were considered lost to follow‐up. From the 381 subjects that came, 166 (43.6%) gained full recovery and were no longer stunted (HAZ > −2.0) in an average of 3.4 months. The effectiveness of stunting management in regional hospitals was 43.6%.

Details of the number of stunted subjects before and after the intervention, along with the percentage of healthcare center visit frequencies, are shown in Table 5. Nagekeo, Kudus, and Sumba Barat Daya were the top three regencies with the greatest decline in stunting rates (7.14%, 6.07%, 4.83%, respectively). An association analysis between Posyandu visit frequency and the rate of stunting decrease revealed that a reduction in stunting is significantly associated with a higher visit frequency (minimum 4 visits in a row; OR 6.8; 95% CI 3.0–15.4; *p* < 0.05), shown in Table 6.

**TABLE 5:** Comparison of stunting prevalence baseline and endline in every region.

No	Region	Stunting baseline	Stunting new cases	Stunting endline	Under‐five children	Stunting prevalence baseline	Stunting prevalence endline	Stunting decrease	*p* *	Coverage of routine weight measurement (Mar–Sep) (%)	PHC visit (%)	Hospital visit (%)
1	Nagekeo	8	0	4	56	14.29%	7.14%	7.14%	0.659	81.48	83.33	100
2	Kudus	75	9	24	247	30.36%	24.29%	6.07%	0.613	80.8	20.54	70.67
3	Sumba Barat Daya	62	2	9	145	42.76%	37.93%	4.83%	0.304	95.82	93.33	61.29
4	OKU Timur	59	16	28	268	22.01%	17.54%	4.48%	0.193	92.05	30.99	50.85
5	Kolaka	61	0	10	266	22.93%	19.17%	3.76%	0.289	65.31	83.33	77.05
6	Purbalingga	16	4	8	119	13.45%	10.08%	3.36%	0.317	93.88	100	90
7	Muara Enim	83	20	31	344	24.13%	20.93%	3.20%	0.193	62.46	41.98	42.35
8	Tulungagung	10	1	5	138	7.25%	4.35%	2.90%	0.42	94.07	83.67	80
9	Kotawaringin	35	6	10	145	24.14%	21.38%	2.76%	0.262	96.94	100	100
10	Pasaman	33	2	5	118	27.97%	25.42%	2.54%	0.222	83.21	23.68	69.7
11	Magetan	35	11	13	219	15.98%	15.07%	0.91%	0.13	95.22	72.73	85.71
12	Blitar	107	29	22	837	12.78%	13.62%	−0.84%	0.125	98.34	32.23	33.64
13	Sragen	18	17	10	223	8.07%	11.21%	−3.14%	0.575	82.99	81.1	77.78
14	Sigi	73	35	19	247	29.55%	36.03%	−6.48%	0.156	73.12	20.59	33.33

*Note:* *Based on chi‐square test.

**TABLE 6:** Association of visit frequency and stunting rate decline.

Visit frequency	Under‐five children	Stunting children				New stunt‐ing case	Stunting case endline total (c)	Change of stunting rate	OR a‐b (95% CI)	OR a‐c (95% CI)
		Baseline (a)	%	Endline (b)	%					
6‐7×	2412	466	19.32%	444	18.41%	97	119	−0.91	1.5 (1.1–2.0)*	3.6 (1.7–7.8)*
4‐5×	450	103	22.89%	104	23.11%	41	40	0.22	2.0 (1.4–2.8)*	6.8 (3.0–15.4)*
2‐3×	501	57	11.38%	65	12.97%	15	7	1.59	Ref	

* Significant with *p* < 0.05 based on chi‐square.

## 4 Discussion

### 4.1 Milk and Egg Supplementation as an Intervention at the Community Level

Studies exploring the addition of animal proteins through milk supplementation for physical growth date back as early as 1991, in which Walker et al. (1991) supplemented 129 Jamaican children aged 9‐ to 24‐month‐old with high‐protein milk‐based supplementations for 6 months and found a significant increase in length by 0.89 cm higher than the control group. Dairy products such as cow's milk or eggs serve as an easy, relatively cheap, and readily available source of animal proteins. The average protein content of 100 mL cow's milk and 1 chicken egg is 3.35 g and 6.3 g, respectively (Antunes et al. 2022; Puglisi and Fernandez 2022). For these reasons, they are often used as interventions in studies exploring the role of animal proteins for child growth.

This study highlights the role of local animal‐source proteins, specifically milk and egg, as a preventive measure against stunting. Proteins contain amino acids that play roles in human growth and metabolism, such as hormone synthesis (growth hormone, insulin‐like growth factor‐1 [IGF‐1], and thyroid hormone), cell membrane protein transporter or receptor, and long bone and joint formation (Joint WHO/FAO/UNU Expert Consultation 2007). The role of essential amino acids, especially lysine, leucine, and tryptophan, for children's linear growth and cognitive development has been scientifically proven in prior studies (Jenkins et al. 2016; Semba et al. 2016; Uauy et al. 2015). The human body cannot produce these essential amino acids, and thus, they need to be acquired from foods. Although plants can be a source of essential amino acids, animal proteins are better because animal proteins contain more complete essential amino acids and have higher bioavailability compared to plant sources (Parikh et al. 2022). A recent systematic review has shown that the deficiency of essential amino acids is commonly found in children in low‐ to middle‐income countries (Dasi et al. 2019). This is especially true in Indonesia, where staple foods, especially rice, are the main source of consumed foods (Diana et al. 2022).

Data from the Ministry of Health Republic of Indonesia showed that 31.9% of under‐five Indonesian children were protein‐deficient (consumed < 80% of the recommended daily protein intake) (Ministry of Health Republic Indonesia 2018). The recommended daily allowance (RDA) of protein for under‐five children in Indonesia is 15 g/day for 6 to 11 months old, 20 g/day for 1 to 3 years old, and 25 g/day for > 3 years old (Ministry of Health Republic Indonesia 2019).

Stunting is preceded by acute malnutrition conditions such as weight faltering or underweight (Isanaka et al. 2019). The first level of stunting prevention should target healthy children at the community level. Our study found that animal protein‐based supplementation using milk and egg in healthy, nonmalnourished children < 5 years old successfully prevented weight faltering in 71.7% of subjects in 6 months. Similar to our findings, a study by Ara et al. (2022) in Bangladesh found that supplementing egg/milk‐based snacks to healthy children aged 6–12 months old, along with multiple micronutrient powder (MNP) and education on child feeding and handwashing for 1 year, successfully reduced stunting by 73%. A recent study conducted in Bangladesh by Mahfuz et al. (2020) gave daily supplementation of one egg, 150 mL of cow milk, and one sachet of MNP for 12‐ to 18‐month‐old children with HAZ scores < 1 for 60 days. Their study found a significant increase in HAZ of the intervention group by 0.23 SD, supporting the role of animal‐source foods in improving the linear growth of children with stunting. Furthermore, Iannotti et al. (2017) also studied the effect of giving daily egg supplementation to healthy 6‐ to 9‐month‐old children (mean HAZ at baseline: −1.90 SD) for 6 months, and found a significant increase in HAZ score by 0.63 SD and a decrease in stunting prevalence by 47%. While the rate of stunting reduction varies in each study, all of these previous studies successfully showed an increase in HAZ following intervention using animal protein source foods. This study showed that subjects who received milk and egg supplementation successfully remained in normal nutritional status throughout the intervention. Further analysis, including the increment in HAZ scores, is ongoing for a future publication. Altogether, it can be concluded that animal proteins have a significant effect on increasing linear growth. Our study strengthens data from previous studies and successfully proves that animal proteins can prevent stunting.

Aside from giving milk and egg supplementation, parents of all subjects initially received education about proper feeding practices, emphasizing the importance of animal proteins as part of their children's daily diets at home. Knowledge and education of the primary caregivers, usually mothers, are well‐known to be important risk factors for stunting (Huriah and Nurjannah 2020). A cross‐sectional study by Yunitasari et al. (2022) involved 502 800 mothers in Indonesia and found that parents' education level and family economic conditions were the top two factors that influence the complementary feeding practices of their children. Mahmudiono et al. (2018) also found that the education of mothers regarding animal protein intake for children resulted in a significant increase in child height and weight from baseline (*p* < 0.001) and a notable improvement in the mothers' behavior in providing more animal protein to the children. These existing findings support the pivotal role of education as part of children's malnutrition management strategies. Education of parents and caregivers at each healthcare level was an integral component that influenced the successful outcomes of this study.

### 4.2 Management of Weight Faltering, Underweight, and Moderate Wasting Children in Primary Care Settings

In most children, weight faltering starts to occur at 3 months old. A study by Emond et al. (2007) has shown that children with weight faltering had significantly lower intelligence quotient (IQ). In addition, Waber et al. (2014) proved that infants with moderate or severe malnutrition also have a ninefold increased risk of having a low IQ < 70 compared to healthy infants. These studies show that cognitive deterioration starts even before stunting occurs, emphasizing the need for early prevention, detection, and management of undernutrition in young children. Therefore, GPs at primary healthcare centers hold a pivotal role in the early detection and prompt treatment of acute malnutrition before a child becomes stunted.

In our study, children who were referred to primary Puskesmas were screened for possible underlying infections and treated accordingly. In addition, a special dietary intervention was given for 2 weeks. Our study found that this intervention in primary healthcare settings can effectively treat weight faltering in 75.6% of subjects. The effectiveness of treatment in primary healthcare settings for subjects with underweight and moderate wasting was 51.9% (41 of 79 subjects recovered) and 64.6% (62 of 96 subjects recovered), respectively. The results of our study highlight the potential solution to the need for early detection, prevention, and management of undernutrition in young children.

### 4.3 Management of Stunting in Secondary Health Care Settings

While it is true that most efforts should be directed toward stunting prevention strategies, it is equally important to properly manage children who are already stunted. In this study, stunting cases in the regional hospitals received FSMP treatment in the form of ONS as indicated. FSMP is a specially developed formula for patients with specific medical conditions or various dietary needs, such as providing nutritional support for catch‐up growth in weight faltering, malnutrition, and premature infants, and its use must be supervised by medical professionals. It is formulated in two forms, that is, oral nutritional supplement (ONS) and to be used via tube feeding for enteral administration (Ruthsatz et al. 2022). According to the Regulation of The Indonesian Food and Drug Authority (2022), the minimum energy density of FSMP for catch‐up growth was regulated to be 0.9 kcal/cc, which is used for stunting management in this study. In this study, we developed a protocol of FSMP guidelines using ONS for children with stunting (Table 4). We found that ONS successfully recovered stunting in 43.6% of stunted subjects. The duration in which subjects reached catch‐up growth varies, with a mean duration of 3.4 months.

A similar study was conducted by Shim et al. (2020), in which they intervened 1 to 3‐year‐old children with growth faltering with 400 mL of oral nutrition supplement containing 1 kcal/cc per day for 6 months. ONS was found to have a significant increase in weight and WAZ score after 6 months (*p* < 0.05) and concluded that an effective catch‐up growth intervention for weight‐faltering children includes concentrated nutritional formula and dietary education. A systematic review and meta‐analysis by Zhang et al. (2021) included 11 RCTs and showed that intervention with ONS for undernourished children resulted in a significantly higher energy intake compared to the control group that received only dietary counseling, placebo, or regular diet. Significant weight and height increases (*p* < 0.05) were also evident in the intervention group after 6 months. Three of the studies showed that weight gain was observed as early as 7–10 days following ONS intervention (*p* < 0.001). Meanwhile, five RCTs reported that height increments were evident after 30–90 days of intervention (Zhang et al. 2021). These existing findings support the results of our studies and highlight the impact of FSMP on catch‐up growth in children with stunting.

### 4.4 Study Implications

The latest stunting data in Indonesia reported a stunting prevalence of 21.6% in 2022 (Ministry of Health Republic Indonesia 2022a). The Ministry of Health Republic of Indonesia has set a stunting reduction target to 14% by 2024 (Ministry of Health Republic Indonesia 2020); thus, a reduction of approximately 7.6% is required to reach this goal. This study demonstrated that our protocol has successfully prevented stunting by 71.7% at the community level and 75.6% in primary healthcare settings using milk and egg supplementation as well as prompt management of red flags in children's growth. The protocol developed in this study has also successfully recovered stunting in 43.6% of subjects through adequate management of stunting using FSMP.

As shown in Table 5, the three regencies with the greatest decline in stunting rates had remarkable primary healthcare and hospital visits. Additionally, increased frequency of visits was associated with a decrease in stunting rates (Table 6). Low compliance was notably observed among study subjects in attending the referral to district hospitals. This may be attributed to logistical and physical barriers as outlined in the WHO conceptual framework of childhood stunting (World Health Organization 2014b). These barriers, including financial constraints and accessibility issues, such as transportation limitations and distance to the healthcare facilities (Beal et al. 2018), likely hindered subjects from fully engaging in the study despite our concerted efforts to encourage participation.

By identifying and addressing compliance challenges among stunted children, this study lays the groundwork for optimizing healthcare system policies. Implementation of the protocols outlined in this study could potentially reduce stunting prevalence by up to 9.4% within 6 months from the rate of 21.6% in Indonesia. This reduction could only be achieved if stunting prevention protocols in primary and secondary healthcare settings were conducted appropriately and the referral system worked effectively, thereby potentially leading to nearly zero new cases of stunting. Therefore, this protocol can help to reach the Indonesian government's stunting reduction goal in as early as 6 months.

This study found that the keys to effectively combat stunting involve a comprehensive strategy that includes: (1) education to caregivers about the importance of animal proteins, (2) early nutritional intervention using locally available animal protein‐rich foods, (3) early detection through routine anthropometric monitoring, (4) early referral to primary health care doctors, (5) early detection of red flags and providing prompt treatment, and (6) targeted nutritional interventions using FSMP for catch‐up growth. The approach to stunting prevention and management should be done systematically, following a tiered referral system according to doctors' competencies. Diagnosing stunting is one of the GP's competencies, while treating stunting is within the competence of a pediatrician. Furthermore, to the best of the author's knowledge, no prior studies have investigated the role of a referral system in stunting, and our study is the first to utilize a tiered referral system as part of stunting prevention and management while also investigating the role of animal protein and FSMP within the referral framework.

## 5 Limitations

Despite the significant contributions of this study, this study is subject to several limitations. This study did not involve a control group, and thus, the comparison between the intervention group and the control group could not be assessed. Since it would be unethical to withhold treatment from a control group, the study design employed a pre–post intervention approach to address this issue. Moreover, with higher compliance, the results of this study might have been more robust. Additionally, our study did not analyze other social factors that might influence the results of this study, such as local government support.

## 6 Conclusion

A comprehensive strategy combining education, community‐based early nutritional intervention, early detection with timely referral, as well as targeted nutritional interventions using FSMP for catch‐up growth prescribed by pediatricians, is needed to effectively reduce stunting prevalence. To conclude, this study demonstrates a promise of substantial stunting reduction within 6 months: 71.7% in the community level, 75.6% in primary healthcare centers, and 43.6% in secondary healthcare centers, underscoring the potential benefits of implementing this protocol to reduce stunting on a larger scale.

## Author Contributions

**Damayanti Rusli Sjarif:** conceptualization (lead), data curation (lead), formal analysis (lead), funding acquisition (lead), investigation (lead), methodology (lead), project administration (lead), resources (lead), supervision (lead), validation (lead), writing – original draft (lead), writing – review and editing (lead). **Klara Yuliarti:** data curation (equal), formal analysis (equal), investigation (equal), methodology (equal), project administration (equal), resources (equal), supervision (equal), validation (equal), writing – original draft (equal), writing – review and editing (equal). **Lanny Christine Gultom:** data curation (equal), investigation (equal), project administration (equal), resources (equal), supervision (supporting). **Cut Nurul Hafifah:** data curation (equal), investigation (equal), project administration (equal), resources (equal), supervision (supporting). **I. Gusti Lanang Sidiartha:** data curation (equal), investigation (equal), project administration (equal), resources (equal), supervision (supporting). **Meta Herdiana Hanindita:** data curation (equal), investigation (equal), project administration (equal), resources (equal), supervision (supporting). **Neti Nurani:** data curation (equal), investigation (equal), project administration (equal), resources (equal), supervision (supporting). **Aidah Juliaty:** data curation (supporting), investigation (equal), project administration (equal), resources (supporting). **Ali Alhadar:** data curation (supporting), investigation (equal), project administration (equal), resources (supporting). **Anik Puryatni:** data curation (supporting), investigation (equal), project administration (equal), resources (supporting). **Arief Budiarto:** data curation (supporting), investigation (equal), project administration (equal), resources (supporting). **Endy Paryanto Prawirohartono:** data curation (supporting), investigation (equal), project administration (equal), resources (supporting). **Irma Sri Hidayati:** data curation (supporting), investigation (equal), project administration (equal), resources (supporting). **Julius Anzar:** data curation (supporting), investigation (equal), project administration (equal), resources (supporting). **Moretta Damayanti:** data curation (supporting), investigation (equal), project administration (equal), resources (supporting). **Nur Aisiyah Widjaja:** data curation (supporting), investigation (equal), project administration (equal), resources (supporting). **Nice Rachmawati Masnadi:** data curation (supporting), investigation (equal), project administration (equal), resources (supporting). **Rina Pratiwi:** data curation (supporting), investigation (equal), project administration (equal), resources (supporting). **Rini Andriani:** data curation (supporting), investigation (equal), project administration (equal), resources (supporting). **Ronald Rompies:** data curation (supporting), investigation (equal), project administration (equal), resources (supporting). **Novitria Dwinanda:** data curation (supporting), investigation (equal), project administration (equal), resources (supporting). **Winra Pratita:** data curation (supporting), investigation (equal), project administration (equal), resources (supporting). **William Jayadi Iskandar:** formal analysis (equal), methodology (equal), writing – original draft (equal), writing – review and editing (equal). **Jessy Hardjo:** formal analysis (equal), methodology (equal), writing – original draft (equal), writing – review and editing (equal).

## Conflicts of Interest

The authors declare no conflicts of interest.

## Supporting information


**Table S1:** List of districts, villages, and Posyandu involved in this study.

## Data Availability

Data described in the manuscript will be made available upon request.
