# Supplementary material for: Effectiveness of a Tiered Referral System and Early Nutritional Intervention to Prevent and Recover Stunting in Under‐Five Indonesian Children
Source: Food Sci Nutr. 2025 Oct 23;13(10):e70945. doi: 10.1002/fsn3.70945 (PMC12547623; doi:10.1002/fsn3.70945)
Supplement: Supplementary file 1 — Table S1: List of districts, villages, and Posyandu involved in this study. [file FSN3-13-e70945-s001.docx]

**Supplementary Table 1.** List of districts, villages, and Posyandu involved in this study

| **Districts** | **Village name** | **Posyandu** |
| --- | --- | --- |
| Pasaman | Tabek | 1. Cempaka 2  2. Cempaka 3 |
| Blitar | Sukosewu | 1. Sukoreno 1  2. Sukoreno 2  3. Sukoreno 3  4. Sukoreno 4  5. Sukoreno 5  6. Sukoreno 6  7. Sukosewu 1  8. Sukosewu 2  9. Sukosewu 3  10. Sukosewu 4  11. Sukosewu 5  12. Sukosewu 6 |
| Tulungagung | Macanbang | 1. Posyandu 1 Dusun Gajah  2. Posyandu 2 Dusun Krajan  3. Posyandu 3 Dusun Trate |
| Magetan | Jabung | 1. Sakura I Setro  2. Sakura II Bulusari  3. Sakura III Gondang  4. Sakura IV Jabung  5. Sakura V Karang |
| Kolaka | Ulukolaka | 1. Darmawulan  2. Anggrek 2  3. Bonsai |
| Muara Enim | Tanjung Agung | 1. Merpati 1 (village 1 & 2)  2. Merpati 2 (village 3 & 4)  3. Merpati 3 (village 5 & 6) |
| OKU Timur | Tugu Mulyo | 1. Melati  2. Mawar  3. Anggrek |
| Purbalingga | Karangaren | 1. Margomulyo 1  2. Margomulyo 2 |
| Sragen | Wonorejo | 1. Mugi Rahayu 1  2. Mugi Rahayu 2  3. Mugi Rahayu 3  4. Mugi Rahayu 4  5. Mugi Rahayu 5  6. Mugi Rahayu 6  7. Mugi Rahayu 7  8. Mugi Rahayu 8 |
| Nagekeo | Woewutu | 1. Kolija  2. Kotapapa  3. K. Baru |
| Sumba Barat Daya | Weekambala | 1. Rajawali  2. B. Karmel  3. Ikan Layang |
| Kudus | Glagahwaru | 1. Anggrek 3  2. Bugenvil 1  3. Bugenvile 6.2  4. Kamboja 4  5. Mawar 2  6. Melati 5 |
| Sigi | Sigi | 1. Kamaipura I  2. Kamaipura II  3. Kamaipura III |
| Kotawaringin Timur | Handil Sohor | 1. Tirtasari  3 additional weigh stations |
